# Diversity of *Eimeria* spp. in dairy cattle of Guwahati, Assam, India

**DOI:** 10.14202/vetworld.2015.941-945

**Published:** 2015-08-07

**Authors:** M. Das, D. K. Deka, P. C. Sarmah, S. Islam, S. Sarma

**Affiliations:** 1 ICAR Research Complex for NEH Region, Barapani, Meghalaya, India; 2 Department of Parasitology, College of Veterinary Science, Guwahati, Assam, India; 3 Department of Biochemistry, C.V.Sc, Guwahati, Assam, India

**Keywords:** Assam, dairy cattle, *Eimeria* spp, prevalence

## Abstract

**Aim:**

To determine the prevalence and diversity of *Eimeria* spp. in dairy cattle present in and around Guwahati, Kamrup district, Assam, India.

**Materials and Methods:**

A total of 2339 fecal samples of calves (535), heifer (641) and adult (1163) cattle were screened for 1 year present in and around Guwahati, Assam for detection of *Eimeria* oocysts by flotation techniques. Sporulation of the oocyst was done in 2.5% potassium dichromate solution for identification of the *Eimeria* species.

**Results:**

Examination of fecal samples revealed an overall prevalence of 11.97% *Eimeria* infection in dairy cattle of Guwahati, Assam. Age-wise, 33.2%, 45.4%, and 21.4% infections were recorded in calves (<1 year), heifer (1-3 years) and adult (>3 years) cattle, respectively. Season-wise, infection was recorded highest during post-monsoon (16.29%), followed by monsoon (15%), winter (9.44%), and pre-monsoon (7.49%) season. Seven species of *Eimeria* were recorded *viz*. *Eimeria bovis*, *Eimeria zuernii*, *Eimeria subspherica*, *Eimeria bukidnonensis*, *Eimeria auburnensis*, *Eimeria ellipsoidalis* and *Eimeria alabamensis*. The oocyst count per gram of feces ranged from 50 to 1500 in infected cattle.

**Conclusion:**

This study indicates that there is the prevalence of seven species of *Eimeria* in dairy cattle of Guwahati, Assam and mostly prevalent during the post-monsoon season.

## Introduction

Coccidiosis is one of the most pathogenic intestinal diseases caused by different species of *Eimeria* belonging to phylum-apicomplexa [1]. They are responsible for huge economic losses to the livestock industry in terms of mortality and morbidity in young calves [2,3]. The disease is particularly a problem of confined animals kept under intensive husbandry practices and is more common in housed animals than in those on pastures. In associations with other enteropathogens, coccidia have been indicated as an important cause of diarrhea in calves [4].

The disease occurs in acute, subacute and chronic forms. Bloody diarrhea, dehydration, rough hair coat, reduced growth rate, anemia, weakness and weight loss are the signs of coccidiosis [5]. Clinical coccidiosis in cattle mainly depends on factors like species of *Eimeria*, the age of the infected animal, the number of oocysts ingested, the presence of concurrent infections and management practices [6]. Overcrowding and lack of sanitation increase the chance of infection. More than 13 species of *Eimeria* and one species of *Isospora* have been described to infect cattle. *Eimeria bovis* and *Eimeria zuernii* are the most pathogenic species and associated with clinical coccidiosis under field conditions while other species have been shown to be mildly or moderately pathogenic. The major damage is due to the rapid multiplication of the parasite in the intestinal wall, and the subsequent rupture of the cells of the intestinal lining. Several stages of multiplication occur before the final stage, the oocyst, is passed in the feces. Oocysts are extremely resistant to environmental stress and are difficult to completely remove from the environment.

The disease is transmitted by ingestion of sporulated oocysts. Infection is acquired from contaminated feed, water, soiled pastures or by licking contaminated hair coat. Therefore, taking into account the significance of the parasite as one of the most important causes of economic losses, the present study was designed to determine the prevalence and diversity of *Eimeria* spp. in dairy cattle of Guwahati, Assam.

## Materials and Methods

### Ethical approval

The experiments comply with the guidelines laid down by the Institutional Ethical Committee and in accordance with the country law.

Samples were collected as per standard collection procedure without harming or any discomfort to animals.

### Study area

The present study was conducted in Guwahati, the capital city of the state of Assam, that lies within the latitude of 26°11’0″N and longitude 91°44’0″E. The city is situated on an undulating plain with varying altitudes of 49.5-55.5 m above mean sea level. The southern and eastern sides of the city are surrounded by hillocks.

### Study period

The study was conducted for one calendar year from August 2012 to July 2013 and divided into four seasons *viz*. Pre-monsoon (March, April, and May), Monsoon (June, July, August, and September), Post-monsoon (October, November) and Winter (December, January, February).

### Sample size

A total of 2339 fecal samples of calves (535), heifer (641) and adult (1163) cattle were collected from both Government and Private farms and screened for detection of *Eimeria* infection in Guwahati, Kamrup district, Assam.

### Study method

The selected animals were categorized according to age *viz*. calves (<1 year), heifer (1-3 years) and adult (>3 years). Fecal samples were collected directly from the rectum of the individual animal and kept in marked plastic pouch/vials. Three grams of fecal samples were examined by direct flotation technique using saturated salt (specific gravity: 1.20) and sucrose (specific gravity: 1.27) solution [7]. Positive samples were then quantified to estimate the oocysts per gram (OPG) of feces by using modified McMaster technique [8]. Samples not being examined on the same day were preserved in 2.5% potassium dichromate solution and stored at refrigerated temperature (4°C) for next day examination. Sporulation of the oocyst was done by mixing positive fecal sample containing oocyst of *Eimeria* spp. with 2.5% potassium dichromate solution in a ratio of 1:5 volume as per the procedure described by Duszynski and Wilber [9] and incubated at room temperature for 4-7 days, checked daily. Morphological characterization and measurement of oocysts was done as per the guidelines of Duszynski and Wilber [9] and Soulsby [10] by using an Olympus BX51 light microscope at ×200 and ×400 magnifications. Microphotographs of the oocysts were taken by using a digital camera (Sony DSC: WX80/B). Micrometry of the oocyst was done as per the procedure described by Sloss *et al*. [11] and 20 oocysts of each species were measured and identified.

### Statistical analysis

Data were statistically analyzed using Chi-square tests for significance using SPSS 15 version. (SPSS Inc., 233 South Wacker Drive, 11^th^ Floor, Chicago, IL 60606-6412)

## Results and Discussion

**Figure-1:**
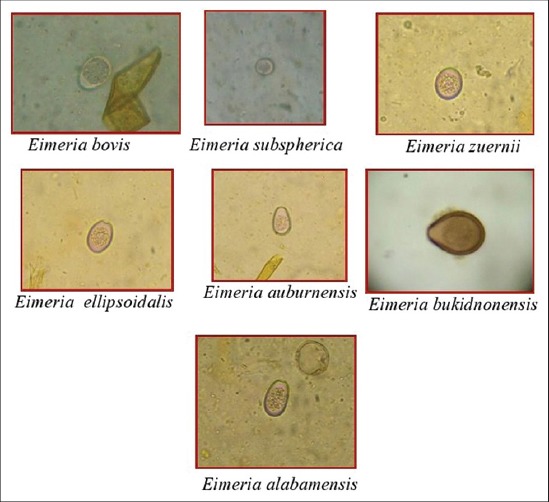
Different oocysts of *Eimeria* species of cattle (400×).

The overall prevalence of coccidiosis in dairy cattle was 11.97% (Table-1). Seven species of *Eimeria* were recorded *viz*. *E. bovis* (6.80%), *E. zuernii* (2.35%), *Eimeria subspherica* (0.68%), *Eimeria bukidnonensis* (0.94%), *Eimeria auburnensis* (0.86%), *Eimeria ellipsoidalis* (0.13%) and *Eimeria alabamensis* (0.21%). All the species of *Eimeria* were identified on the basis of their morphological characters (Figure-1). The length × width (mean±standard error) of each species were *E. subspherica* (10.1±0.46×9.8±0.69 µm), *E. ellipsoidalis* (15.1±0.57×12.2±0.68 µm), *E. zuernii* (16.4±0.43×14.3±0.35 µm), *E. alabamensis* (17.2±0.27×11.1±0.31 µm), *E. bovis* (25.4±0.52×19.4±0.72 µm), *E. auburnensis* (36.4±0.34×21.2±0.47 µm) and *E. bukidnonensis* (43.4±0.23×30.1±0.48 µm).

**Table-1:** Prevalence of eimerian infection in dairy cattle of Guwahati, Assam.

Season	Sample screened	*E. bovis*	*E. zuernii*	*E. subspherica*	*E. bukidnonensis*	*E. auburnensis*	*E. ellipsoidalis*	*E. alabamensis*	Total positive sample
Pre-monsoon	574	27 (62.79)	3 (6.97)	6 (13.95)	3 (6.97)	3 (6.97)	-	1 (2.32)	43 (7.49)
Monsoon	773	68 (58.62)	26 (22.41)	6 (5.17)	9 (7.75)	5 (4.31)	-	2 (1.72)	116 (15)
Post-monsoon	399	33 (50.76)	14 (21.53)	4 (6.15)	7 (10.76)	7 (10.76)	-	-	65 (16.29)
Winter	593	31 (55.35)	12 (21.42)	-	3 (5.35)	5 (8.92)	3 (5.35)	2 (3.57)	56 (9.44)
Overall	2339	159 (6.80)	55 (2.35)	16 (0.68)	22 (0.94)	20 (0.86)	3 (0.13)	5 (0.21)	280 (11.97)

Figures in parentheses indicates percentage. *E. bovis=Eimeria bovis, E. zuernii=Eimeria zuernii, E. subspherica=Eimeria subspherica, E. bukidnonensis=Eimeria bukidnonensis, E. auburnensis=Eimeria auburnensis, E. ellipsoidalis=Eimeria ellipsoidalis, E. alabamensis=Eimeria alabamensis*

**Figure-2:**
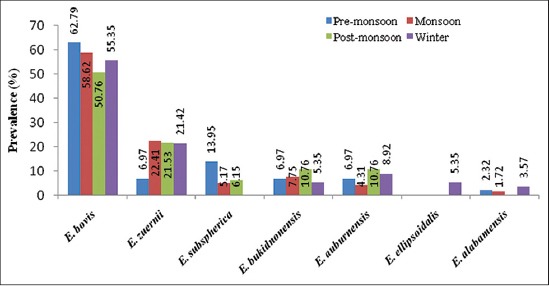
Seasonal prevalence of different eimerian species in cattle of Assam.

**Figure-3:**
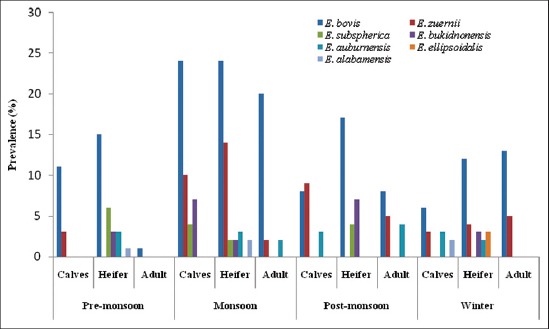
Seasonal prevalence of eimerian species in different age groups of cattle.

Season-wise infection was recorded highest during post-monsoon (16.29%) followed by monsoon (15%), winter (9.44%) and pre-monsoon (7.49%) season (Table-1, Figures-2 and -3). During monsoon season high prevalence of coccidia infection in calves was also reported from Parbhani, Maharastra [12]. High prevalence during monsoon and post-monsoon seasons may be due to favorable conditions such as optimal moisture, humidity, and temperature for easy dispersion, sporulation and transmission of *Eimeria* sp. The peak prevalence of *Eimeria* spp. infection in the rainy season could also be attributed to the presence of precipitating stress, inclement weather, wet conditions and the highest rate of faecal contamination of feed and water [13]. It might be also due to the non-administration of coccidiostat or coccidicidal drugs by the farmers or veterinarians that do not reach the rural population thus maintaining a uniform infection throughout the year. In Poland, seasonal variation in shedding of *Eimeria* oocysts in European Bison and highest prevalence was noted in early spring, with a peak in April, and the lowest in late autumn and winter [14].

In the present findings, it has been observed that the prevalence of *Eimeria* infection followed an age pattern. The infection rate was highest in heifer (45.4%), followed by calves (33.2%) and adult (21.4%). Chi-squared test revealed significant difference (p<0.05) in the pattern of prevalence according to age (Table-2). In heifer, the species recorded were of *E. bovis*, *E. zuernii*, *E. subspherica*, *E. bukidnonensis*, *E. auburnensis*, *E. ellipsoidalis* and *E. alabamensis*. In calves too *E. bovis* was maximum followed by *E. zuernii*, *E. bukidnonensis*, *E. auburnensis*, *E. subspherica* and *E. alabamensis*. However, in adults, only three species i.e. *E. bovis*, *E. zuernii* and *E. auburnensis* were recorded. The OPG of feces ranged from 50 to 1500 in infected cattle (Table-2). The infection rate was observed higher in heifer than calves which may be due to housing of heifer in overcrowded conditions and easy contact with adult animals. Similarly, the higher infection rate in calves >6-12 months of age than calves of 1-6 months of age was observed [15]. Priti *et al*. [16] also observed higher prevalence in younger animals than adult and stated that immature immunity might be a critical factor for determining the clinical and subclinical infections in younger animals. The possibility of adult animals acting as a reservoir for younger ones in stall fed conditions is also an added explanation [17].

**Table-2:** Diversity of *Eimeria* sp. in different age groups of cattle.

Eimeria spp.	Calves		Heifer		Adult		Chi-square value
		
	Prevalence (%)	OPG of faeces	Prevalence (%)	OPG of faeces	Prevalence (%)	POG of faeces	
*E. bovis*	49 (52.7)	50-1200	68 (53.5)	50-1500	42 (70)	50-900	26.61*
*E. zuernii*	25 (26.9)	50-700	18 (14.2)	50-900	12 (20)	50-400	
*E. subspherica*	4 (4.3)	50-500	12 (9.4)	50-700	-	-	
*E. bukidnonensis*	7 (7.5)	50-150	15 (11.8)	50-400	-	-	
*E. auburnensis*	6 (6.5)	50-200	8 (6.3)	50-500	6 (10)	-	
*E. ellipsoidalis*	-	-	3 (2.4)		-	-	
*E. alabamensis*	2 (2.2)	50-100	3 (2.4)	50-200	-	-	
Overall	93 (33.2)	50-1200	127 (45.4)	50-1500	60 (21.4)	50-900	

* p<0.05,

-=Negative, OPG: Oocyst per gram, *E. bovis=Eimeria bovis, E. zuernii=Eimeria zuernii, E. subspherica=Eimeria subspherica, E. bukidnonensis=Eimeria bukidnonensis, E. auburnensis=Eimeria auburnensis, E. ellipsoidalis=Eimeria ellipsoidalis, E. alabamensis=Eimeria alabamensis*

*E. bovis* and *E. zuernii* accounted for highest prevalent species in the present study which is in conformity with the findings of Heidari *et al*. [18] and Yu *et al*. [19] from Iran and China, respectively. Other species of *Eimeria viz*. *E. bukidnonensis*, *E. subspherica*, *E. auburnensis*, *E. alabamensis* and *E. ellipsoidalis* were also recorded in cattle with varying percentage which is in conformity with the reports from Brazil [1], Pakistan [3], Hungary [20], India [21], Poland [22], and China [23]. The variation in prevalence of *Eimeria* spp. may be attributed due to different geographical distributions, host factors and climatic conditions required for their development. Borkakoty *et al*. [24] also reported prevalence of *E. bovis*, *E. zuernii*, *E. auburnensis*, *E. ellipsoidalis*, *E. cylindrica*, *E. bukidnonensis* and *E. subspherica* in calves and adult cattle from Kamrup district of Assam. The present study was done after a spell of several years in the same region and showed the persistence of infection in animals. Thus, we can conclude that the infection still continues and eimeriosis is not to be neglected in field condition because this infection is opportunistic.

## Conclusion

The present study revealed that there is the prevalence of seven species of *Eimeria* in dairy cattle of Guwahati, Assam, and the prevalence was highest during the post-monsoon season.

## Authors’ Contributions

MD: Collected, processed and examined fecal samples, prepared manuscript. DKD: Interpretation of data. SI: Examined samples. PCS, SS: Prepared manuscript and data analysis. All authors read and approved the final manuscript.
